# Populations, pools, and peccaries: simulating the impact of ecosystem engineers on rainforest frogs

**DOI:** 10.1093/beheco/aru243

**Published:** 2015-01-29

**Authors:** Max Ringler, Walter Hödl, Eva Ringler

**Affiliations:** ^a^Department of Integrative Zoology, University of Vienna, Althanstrasse 14, A-1090 Vienna, Austria and; ^b^Department of Cognitive Biology, University of Vienna, Althanstrasse 14, A-1090 Vienna, Austria

**Keywords:** Allobates femoralis, Dendrobatidae, ecosystem engineering, nontrophic interaction, population manipulation experiment, reproductive resource supplementation.

## Abstract

“Ecosystem engineering” describes habitat alteration by an organism that affects another organism; such nontrophic interactions between organisms are a current focus in ecological research. Our study quantifies the actual impact an ecosystem engineer can have on another species by using a previously identified model system—peccaries and rainforest frogs. In a 4-year experiment, we simulated the impact of peccaries on a population of *Allobates femoralis* (Dendrobatidae) by installing an array of artificial pools to mimic a forest patch modified by peccaries. The data were analyzed using a gradual before-after control-impact (gBACI) model. Following the supplementation, population size almost doubled as a result of increased autochthonous recruitment driven by a higher per-capita reproduction of males and a higher proportion of reproducing females. The effect was evenly distributed across the population. The differential response of males and females reflects the reproductive behavior of *A. femoralis*, as only the males use the aquatic sites for tadpole deposition. Our study shows that management and conservation must consider nontrophic relationships and that human “ecosystem engineering” can play a vital role in efforts against the “global amphibian decline.”

## Lay Summary

Peccary wallows and footprints are important breeding pools for rainforest frogs. We performed a resource supplementation experiment with artificial pools, simulating peccary actions, in a population of the poison frog *Allobates femoralis*. The population almost doubled resulting from increased local reproduction, but not from immigration. These findings demonstrate the importance of “ecosystem engineers,” such as peccaries, for other species, the frogs. Our results also indicate that human engineering may help to protect amphibian populations.

## INTRODUCTION

Amphibians are the most endangered terrestrial vertebrates, with well-documented declines especially in the tropics (Alford 2011). Numerous stressors have been identified, and human habitat modification is the single most important driver of decline. Several stressors are related to and amplified by climatic change (Blaustein and Walls 2010). The complex life cycle of amphibians, with aquatic and terrestrial life-history stages in many species, makes them particularly vulnerable to these potentially additive environmental threats (Vonesh and De la Cruz 2002; Hof et al. 2011; Salice et al. 2011). Initially, only certain amphibian species and populations suffered from the “amphibian decline” phenomenon, but subsequently, whole species communities and ecosystems became impacted by the loss of keystone species (Whitfield et al. 2007). As adults and larvae of most amphibians occupy entirely different ecological niches, the loss of a single amphibian species is tantamount to losing two species (Whiles et al. 2006). Besides general and conventional conservation and management practices, such as habitat protection and restoration, several emergency measures have been suggested or implemented. These include ex-situ conservation by captive breeding and translocation (Griffiths and Pavajeau 2008). In a recent review, Shoo et al. (2011) suggested a catalog of technically inspired in-situ management actions to “engineer a future for amphibians under climate change.” These involve modifying and enhancing natural habitats to support and stabilize endangered populations. “Ecosystem engineering,” however, is not strictly an anthropogenic activity: many other organisms can potentially fulfill this role (Jones et al. 1994). Such nontrophic interactions have become a focus of ecological research (Wright and Jones 2006) and are now even integrated into ecosystem network modeling (Baskett 2012; Kéfi et al. 2012). Amphibians have limited physical abilities to alter the environment, generally obtaining more benefits from ecosystem engineering performed by other species than vice versa. For example, frogs use bison (Gerlanc and Kaufmann 2003) and peccary wallows (Zimmerman and Bierregaard 1986) as breeding sites, and tree holes and other phytotelmata also represent such “engineered” resources (Lehtinen 2004). In turn, tadpoles can considerably alter habitat structure and biomass availability in streams and ponds by influencing sediment distribution and algae communities (Flecker et al. 1999; Ranvestel et al. 2004; Whiles et al. 2006; Wood and Richardson 2010). The relevance of ecosystem engineers in conservation management was recognized even before Jones et al. (1994) coined the term. For rainforest anurans, Zimmerman and Bierregaard (1986) argued that peccaries might determine the minimum area needed to preserve species communities, and Reider et al. (2013) showed that collared peccaries positively affect terrestrial frogs in a lowland forest in Costa Rica.

### Reproductive resources in dendrobatid frogs

Complex life cycles often require amphibians to cope with threats and risks in aquatic and terrestrial environments. While lentic eggs and tadpoles (mode 1 sensu Duellman and Trueb 1994) are seen as the primitive mode of reproduction in anurans, frogs have evolved many remarkably diverse responses to specific threats to eggs and tadpoles, the most vulnerable life stages (cf. Crump 1974; Magnusson and Hero 1991; Brown et al. 2010). Most dendrobatid frogs feature a strategy in which eggs are deposited out of the water and hatch into feeding tadpoles. They are then carried to water by one of the parents to complete metamorphosis (mode 14 sensu Duellman and Trueb 1994). Accordingly, the availability and characteristics of sites and substrates for egg deposition, and suitable bodies of water for tadpole development determine the autecology and population ecology of dendrobatid frogs, which has been shown in several studies: In *Oophaga pumilio*, supplementation of leaf litter for egg deposition and bromeliads for tadpole development were found to influence population density by behavioral (Donnelly 1989a) as well as demographic responses (Donnelly 1989b). A study in *Ranitomeya imitator* identified the properties of water bodies as the key ecological trait in the evolution of biparental care, and of social and genetic monogamy (Brown et al. 2010). In *Allobates femoralis*, the location of tadpole deposition sites influences year-to-year displacement of individuals that survive more than one breeding season (Ringler et al. 2009). Male *A. femoralis* opportunistically use terrestrial water bodies ranging from shed palm bracts, peccary wallows, to natural ponds, and larger temporarily flooded areas (Gascon 1995; Ringler et al. 2009; Beck et al. 2010). This indiscriminate use of breeding sites contrasts with the often extremely specialized tadpole-rearing sites of other dendrobatids (cf. Brown et al. 2010). The ephemeral and fluctuating occurrence of suitable water bodies forces male *A. femoralis* to leave their territories to deposit tadpoles, sometimes more than 180 m away (Ringler et al. 2013), from where they reliably return to their home territories (Pašukonis et al. 2013, 2014).

This study summarizes the results of a 4-year population manipulation experiment with a Neotropical poison frog. In an *A. femoralis* population where males had only ephemeral bodies of water available for tadpole deposition, we added an array of artificial pools as a new reproductive resource, using the established approach of Zimmerman and Simberloff (1996) to mimic a forest patch that was modified by peccaries (Tayassuidae) through trampling and wallowing. Peccaries are important ecosystem engineers for Neotropical rainforest frogs by providing breeding sites and dry season habitat (Beck et al. 2010; Reider et al. 2013).

We hypothesized that the supply of additional breeding pools should result in 1) a larger adult population size of *A. femoralis* as a result of: 2) increased autochthonous reproduction; and/or 3) increased adult survival during the dry season; and/or 4) greater immigration from other areas. We assessed the impact of the manipulation on population genetics and individual reproductive success through a pedigree analysis across the 4 cohorts in our study population.

## MATERIALS AND METHODS

Our study was approved by the scientific committee of the research station where fieldwork was conducted. All necessary permissions for toe clipping and sampling of larvae were provided by the “Centre National de la Recherche Scientifique Guyane” (CNRS Guyane, permit numbers: 12/05/2009 and 16/12/2009) and by the “Direction Régionale de l’Environment de Guyane” (DIREN, permit number: arrêté n°/2010–015). All sampling was conducted in strict accordance with current French and EU law and followed the ASAB guidelines (Association for the Study of Animal Behaviour 2012).

### Population and sampling

**Figure 1:**
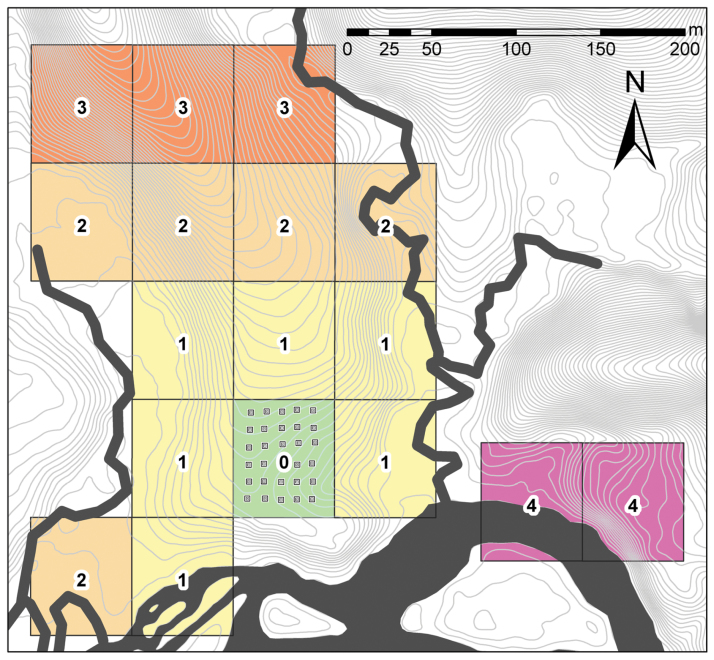
Spatial arrangement of the treatment plot with artificial pools (small squares) and control plots; numbers and color/shading indicate the distance level (1–4) from the treatment plot (0); creeks and the Arataye River in dark gray; elevation lines in light gray.

The *A. femoralis* population is located near the field station “Saut Pararé” (4°02ʺN, 52°41ʺW; WGS84) in the nature reserve “Les Nouragues” in French Guiana. The lowland rainforest here is characterized in detail by Bongers et al. (2001), and the study area and the naturally delimited spatial setup of the *A. femoralis* population are described in detail by Ursprung et al. (2011a). The “study plot” of the latter is termed “study area” in this study, to avoid confusion with the 17 “study plots” that we used here for demographic analyses (Figure 1). Pluviometric data from the closest permanent weather station (Cacao, ~60 km from Pararé) were obtained from the French national meteorological service. Cumulative rainfall in hydrological years (beginning of rainy season to end of dry season; October to September) varied between 3205 and 4081mm from 2006 to 2010, and the driest months, September and October, received on average 71 and 90mm during this time, respectively. Two peccary species potentially occur in the region, *Pecari tajacu* (collared peccary) and *Tayassu pecari* (white-lipped peccary), though the latter has not been sighted in the Nouragues Reserve during the last 10 years (Hibert et al. 2011; Richard-Hansen et al. 2014). Prior to our study, we searched the whole study area for water bodies potentially serving as tadpole deposition sites. We found several depressions close to the bordering river and creeks (Figure 1), which filled up during heavy rains and flooding. However, during our study, none of these pools persisted for more than 5 days. No peccary wallows were present during our study. We concluded that, prior to our experiment, the *A. femoralis* males here had mainly relied on small ephemeral water-filled structures such as palm bracts, nuts, leaves or puddles on fallen tree trunks, as reported previously for this species (Gascon 1995; Ringler et al. 2009; Beck et al. 2010). The population was monitored for 4 seasons (28 January–24 April 2008; 17 January–16 March 2009; 16 January–16 March 2010; 30 January–24 February 2011) with a varying number of coworkers, totaling 150, 227, 200, and 182 people-days in the field, respectively. Surveys took place daily from 0900 to 1900 hours, with an equal time effort spent per unit area per day to ensure equal sampling across time and space within any season. We identified individual frogs manually based on their unique ventral pattern using the pattern matching program Wild-ID (Bolger et al. 2012); sex was determined by the presence (males) and absence (females) of skin folds of the vocal sac. All data were recorded in the field on a highly detailed digital map (Ringler et al. 2014), using pocket computers with the mobile GIS software ArcPad™7/8/10 (ESRI, Redlands, CA), and further handled in ArcGIS^™^9.3.1 (ESRI).

### Resource supplementation experiment

On 17 February 2009, we installed 30 artificial pools (plastic bowls, 30×30×20cm) in a regular 5×6 north–south array with a spacing of 10 m between pools in a central part of the study population (Figure 1). This supplementation used the established design of Zimmerman and Simberloff (1996) to mimic a forest patch with water-filled depressions, which is the characteristic outcome of peccary trampling and wallowing (Beck et al. 2010; Raynaud et al. 2013). The bowls were dug level into the ground and filled by rain. Every bowl was supplied with a branch to allow frogs and other animals to escape. Approximately, 500cm^3^ of soil and leaf litter were added to each pool to provide substrate for tadpoles. The bowls were used for tadpole deposition by *A. femoralis* males immediately after the next rainfall on 20 February 2009. The bowls never dried out because of the typically high rainfall in the Nouragues Reserve, also during the dry season (this study, Bongers et al. 2001).

### Demographic analyses

To analyze the demographic effects of our resource supplementation experiment across space and time, we used a gradient before-after control-impact (gBACI) study design (cf. Ellis and Schneider 1997); the study plots were treated as adjacent, nonindependent subunits of the entire study area. The BACI design is widely employed in studies of single-site impacts, disaster analysis, and generally when the replication of experiments is precluded by logistic, legal, or natural constraints (Stewart-Oaten et al. 1986; Conquest 2000; Smith 2002). We decided for this experimental design and against a fully replicated setup of independent pools across the forest to be able to identify internal population genetic effects of the manipulation together with demographic effects on the entire population.

We partitioned the study area into a treatment plot, which encompassed the artificial pool array along with a surrounding buffer zone of 10 m, and 14 adjacent control plots of equal size (60×70 m). Two additional detached control plots, equally inhabited by *A. femoralis*, were located entirely outside the study area and separated from it by a creek (Figure 1). Only plots where at least 50% of the area was within the study area were considered.

Annual total population sizes and sampling coverages (sampled/estimated individuals) of males and females inside the study area were determined using EstimateS 8.2.0 (Colwell 2006). We used individual mark-recapture histories with the asymptotic population estimator MMMeans, as recommended for our sampling scheme by Brose and Martinez (2004). This yielded estimates of the total number of individuals that had lived in the study area in any given year. Then, we applied a rarefaction analysis (Luikart et al. 2010) to ensure the comparability of the individual-based data in our spatially explicit analyses across years with different sampling coverages. We first determined the highest common sampling coverage across all years of sampling for males and females, respectively. Then, we used the according rarefaction counts (*S*_obs_ (Mao Tau) in EstimateS) to identify those individuals that were sampled in a particular year until these highest common sampling coverages had been reached for males and females, respectively. Individuals were then assigned to the respective study plots based on their center of activity, calculated as the centroid of all encounters of any given individual.

Population size and distribution across space and time were assessed with a repeated measures analysis of variance (Anova) with a general linear model (GLM) in MINITAB™16 (Minitab Inc., State College, PA), with the rarefied numbers of individuals per plot as the response variable. The model contained “treatment” (2 levels; before and after) and “distance” (5 levels; treatment plot-outside plot: 0-4) as main factors, the factor “year” (4 levels; 2008–2011) was nested inside “treatment” and the factor “plot identity” (17 levels) was nested inside “distance.” The factor “sex” (2 levels; male and female) accounted for a probable male bias in the operational sex ratio (cf. Ursprung et al. 2011a). The interaction term “distance × treatment” was added to the model to account for a possible differential effect of the treatment with increasing distance from the treatment plot, and the interaction term “treatment × sex” was added to account for a possible differential male/female response to the resource supplementation. To test for a possible impact of rainfall on population size, cumulative precipitation in the hydrological year that preceded a particular year of sampling was analyzed as a covariate. This test was performed in a repeated measures analysis of covariance (Ancova). As the covariate “previous rainfall” was collinear to the factor “year,” which resulted in a rank deficiency in the estimation of the GLM parameters, we used an altered GLM with the nested factor “year” omitted.

The impact of the manipulation on survival was assessed using the survivor rates (= rarefied number of survivors in a plot/rarefied total number of individuals in the previous year) in the treatment and control plots for males and females, respectively. The data on survivor rates were then used as response variable in the Anova/Ancova analyses.

### Genotyping, parentage assignment, and population genetics

We conducted parentage assignments to investigate which new individuals in a certain year were offspring from individuals that had been sampled in preceding years to assess source-sink-dynamics and migration (cf. Peery et al. 2008). For this pedigree analysis, we used all individuals of the full Pararé population that were encountered inside the study area. Parentage assignments were made between consecutive annual cohorts and were performed with the program COLONY 2 (Wang 2009). COLONY simulates parental genotypes in cases when no matching parent is found among the candidate parents. This feature allowed identifying individuals with parents that had not been sampled inside the study area, either because the parents did not inhabit this area or because they were missed during sampling.

Tissue samples were taken from all individuals by removing the third toe of both hind limbs, a procedure that has no detrimental effect on *A. femoralis* (Ursprung et al. 2011b). DNA was isolated by a proteinase K digestion followed by a standard phenol–chloroform extraction. Seven highly polymorphic microsatellite loci were amplified, run on an ABI 3130×l sequencer, and analyzed using PeakScanner™ 1.0 (Applied Biosystems, Foster City, CA). Details on the microsatellite loci, the PCR protocol, and the subsequent data treatment and pedigree estimation are published in Ursprung et al. (2011a). For most frogs sampled in 2008 and 2009, we used the genotype data from Ursprung et al. (2011a), whereas all frogs from 2010 and 2011 were genotyped for the first time. We computed new parentage assignments across all sampling years to use the most recent version of COLONY (2.0.1.4, at the time of computation) and to use the same spatial sampling unit in all calculations.

## RESULTS

### Demography

From 2008 until 2011, we captured 683 individual males and 291 females of *A. femoralis*. Of these individuals, 471 males and 219 females were found inside the treatment and control plots. The MMMeans estimators indicated a maximum common sampling coverage of 72.6% for males and 51.5% for females inside the plots across the 4 years of sampling. The operational sex ratio, as calculated from the MMMeans estimators, differed significantly (2 proportions *Z* = −4.11, *P* < 0.001) between the pre- (1.66 males/female) and post-treatment years (1.33 males/female). The individual rarefaction counts, derived from the maximum common sampling coverages, were used as population sizes in the Anova and Ancovas to ensure data comparability across all 4 years (Table 1).

**Table 1:** Counts, estimators, and rarefaction for individual males and females in the entire population and in the study plots

	2008	2009	2010	2011
Captured total^a^				
♂	147	160 (22)	204 (6/12)	245 (0/1/32)
♀	60	71 (8)	97 (3/12)	107 (1/5/15)
Captured in study plots^a^				
♂	99	116 (19)	145 (5/5)	163 (0/0/23)
♀	49	53 (8)	76 (3/12)	83 (1/3/15)
Estimate in study plots^b^				
♂	123 (81.2%)	159 (77.7%)	199 (76.2%)	237 (72.2%)
♀	78 (62.7%)	92 (59.6%)	149 (53.2%)	178 (50%)
Rarefaction in study plots				
♂	89	107	137	163
♀	41	46	73	83
Survival rate in study plots				
♂	—	0.21	0.09	0.17
♀	—	0.2	0.32	0.26

^a^Totals include survivors from previous years, which are given in parentheses for 2008/2009/2010.

^b^Percentages indicate sampling coverage.

In 2009, after installing the artificial pools, only 1 additional, previously unsampled individual was found on the treatment plot. The individuals present before in and around the treatment plot showed no apparent reaction, which was corroborated by the stable male territories during intrusion trials performed simultaneously from 28 February–16 March 2009 in the region of the treatment plot (Ringler et al. 2011).

**Figure 2:**
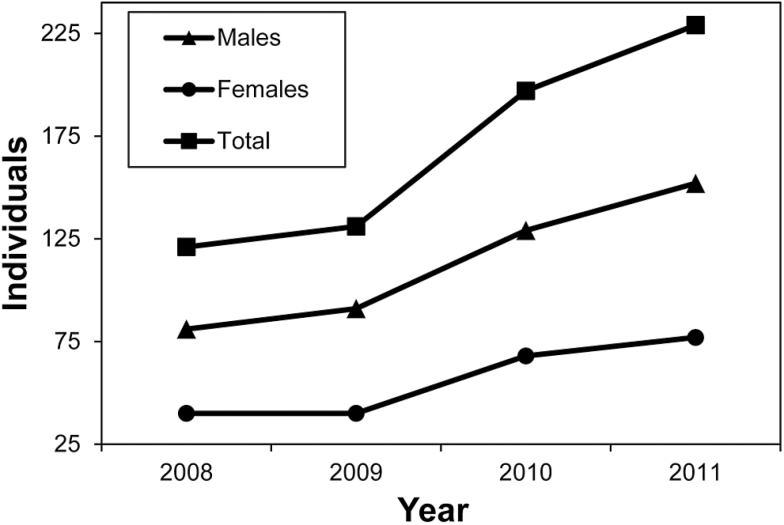
Rarefied counts for males and females inside the treatment and the adjacent control plots.

**Figure 3:**
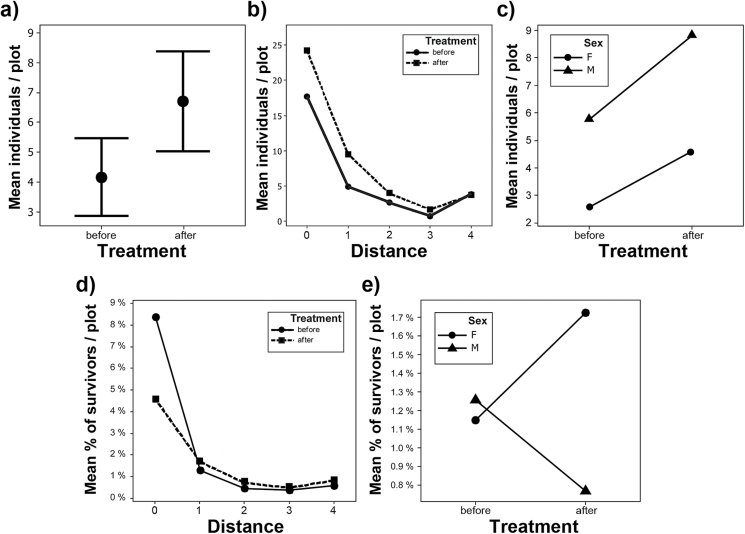
Main effect and interaction plots of the response variable “individuals,” (a) main effect of “treatment,” error bars indicate 95% confidence intervals of means, (b) interaction “treatment” × “distance,” and (c) interaction “treatment” × “sex”; and interaction plots of the response variable “% of survivors,” (d) interaction “treatment” × “distance,” and (e) interaction “treatment” × “sex.”

**Figure 4:**
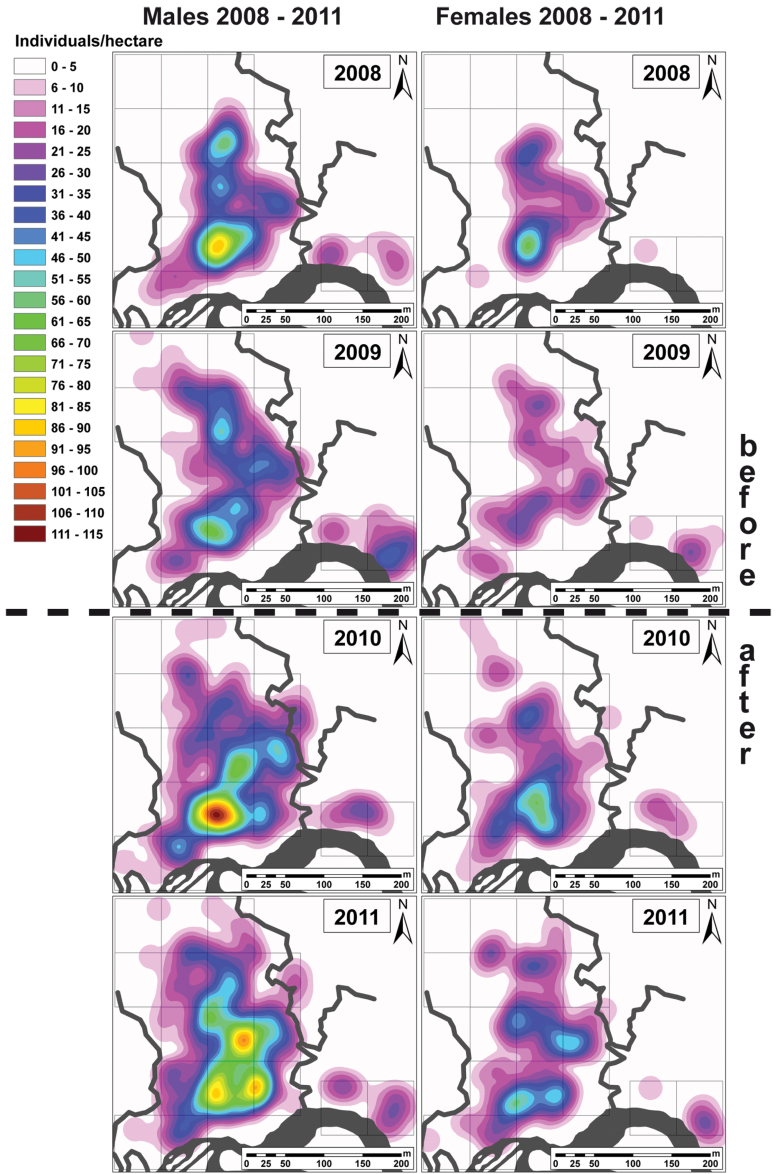
Density maps of males (left) and females (right) across the 4 years of the experiment; rectangles indicate treatment and control plots (cf. Figure 1); colors indicate density of males and females per hectare; kernel density calculated in ArcGIS© 9.3.1 with a search radius of 35 m and a cell size for analysis of 0.2×0.2 m.

The Anova GLM (Table 2, 2a) explained 84.63% (*R* ^2^) of the observed variation in the rarefied counts of individuals in the treatment and control plots. Resource supplementation significantly increased the population size from the pre- to post-intervention years (Figures 2 and 3a): population size almost doubled during the experiment (Table 1, Figures 2 and 4). In contrast, the differences among the pre- and post-treatment years were nonsignificant. The effect of the artificial pools declined significantly with increasing distance from the treatment plot, and the detached control plots were unaffected (Figures 3b and 4). During all years, individual counts varied significantly among plots and were significantly influenced by the distance from the treatment plot (Figure 3b). Males and females had significantly unequal distributions, but there was no significant differential reaction of the sexes to the supplementation (Figure 3c). The Ancova GLM with previous rainfall as a covariate (Table 2, 2b) had a marginally better fit to our data (*R* ^2^ = 84.72%) and, unlike the collinear nested factor “year,” previous rainfall was a significant predictor of population size, whereas the influence of all other factors remained unchanged. The residuals in all Anova/Ancova analyses were normally distributed.

**Table 2:** Anova/Ancova tables of GLMs (a) number of individuals with “year” as a nested factor in “treatment,” (b) number of individuals with the covariate “previous rainfall” instead of the collinear factor “year,” and (c) relative survival with the covariate “previous rainfall”

	a			b			c		
	*R* ^2^ = 84.63%			*R* ^2^ = 84.72%			*R* ^2^ = 53.48%		
Source	df	*F*	*P*	df	*F*	*P*	df	*F*	*P*
Treatment	1	26.01	**<0.001**	1	28.81	**<0.001**	1	1.44	0.234
Distance	4	112.3	**<0.001**	4	112.9	**<0.001**	4	22.6	**<0.001**
Plot (distance)	12	14.79	**<0.001**	12	14.88	**<0.001**	12	3.26	**<0.001**
Year (treatment)	2	2.2	0.116	—	—	—	—	—	—
Treatment × distance	4	5.72	**<0.001**	4	5.75	**<0.001**	14	2.71	**0.036**
Sex	1	76.99	**<0.001**	1	77.45	**<0.001**	11	2.13	0.149
Treatment × sex	1	1.4	0.227	1	1.48	0.223	1	3.35	0.071
Previous rainfall	—	—	—	1	4.07	**0.046**	1	0.01	0.940
Error	110			111			77		
Total	135			135			101		

Significant results at *P* < 0.05 in bold.

### Survivors

The Ancova GLM of the data on survivorship (Table 2, 2c) explained less of the variation in our data (*R* ^2^ = 53.48%) than it did for the rarefied individual counts. There was no significant difference in survivorship before and after pool installation (Table 2, 2c), but the interaction between treatment and distance to the treatment plot was significant: survivors decreased considerably in the treatment plot, but increased slightly in the control plots after installation (Figure 3d). The distance to the treatment plot was a significant factor, as was plot identity. Sex was not a significant factor in the model, the interaction between sex and treatment marginally significant (Table 2, 2c), with female survivors increasing but male survivors decreasing after pool installation (Figure 3e). Previous rainfall was not a significant predictor of survival. Also, the residuals in the Ancova analysis on relative survivorship were normally distributed.

### Parentage analysis

On average, 83.8% of all newly encountered individuals from 2009–2011 could be assigned to parent pairs where at least one partner had been sampled in the preceding year. In each year, all sampled and simulated parents formed a contiguous network of reproductively linked individuals without any detached clusters of entirely simulated parents. There were no significant differences (*Z* = −1.37, *P* = 0.171) in the proportions of offspring with both parents unsampled (= simulated) between pre- (14.4%) and post-treatment years (19.4%). Also, the proportion of simulated individuals in the parent cohorts did not differ significantly (males: *Z* = −0.86, *P* = 0.389; females: *Z* = −1.1, *P* = 0.273) between pre- (males: 22.6%, females: 37.9%) and post-treatment offspring cohorts (males: 30.5%, females: 49.4%). The proportion of reproductively successful males and females among the candidate parents did not differ significantly (males: *Z* = 1.31, *P* = 0.192; females: *Z* = 0.38, *P* = 0.701) between pre- (males: 26.1%, females: 37.5%) and post-treatment offspring cohorts (males: 19.3%, females: 34.4%). The sex ratio of reproductively successful individuals changed from 1.33 males/females pre-treatment to 1.13 males/females post-treatment, but the difference was not significant (*Z* = −1.3, *P* = 0.192).

The number of offspring per father increased significantly (2-sample *T*_53_ = −2.49, *P* = 0.016) from pre- (4.04±1.9 standard deviation [SD]) to post-treatment offspring cohorts (5.3±2.29 SD), whereas the number of offspring per mother decreased from pre- (4±1.33 SD) to post-treatment offspring cohorts (3.95±2.24 SD), but not significantly (*T*_51_ = 0.1, *P* = 0.922). The number of offspring per parent was negatively correlated with the distance to the center of the treatment plot for both sexes. However, these correlations were neither significant for parents of pre- (males: Pearson”s *R* = −0.216, *P* = 0.31; females: *R* = −0.04, *P* = 0.873) nor post-treatment offspring cohorts (males: *R* = −0.085, *P* = 0.557; females: *R* = −0.261, *P* = 0.087).

## DISCUSSION

Our resource supplementation experiment quantitatively demonstrated the impact of simulated peccary presence on anuran population size, suggesting that peccaries can act as ecosystem engineers at our study site. After installing artificial pools to mimic peccary-modified forest, the *A. femoralis* population size almost doubled within only 2 years. Zimmerman and Bierregaard (1986) pointed out the use of peccary wallows by numerous anuran species, and Gascon (1995) described the general advantages of such pools for frogs: both studies explicitly mentioned *A. femoralis*. Recently, Beck et al. (2010) revealed the physical and chemical characteristics of the ecosystem engineering actions of peccaries, identifying the nature of their positive effects on anurans. Their study underlined that the main benefit is a reduced risk of desiccation for the tadpoles. Our study now quantifies the possible population-wide impact of this natural ecosystem engineer on *A. femoralis*, a model species for anuran ecology and behavior.

### Demography

The increase in population size spread gradually across the study area: Control plots near the treatment plot showed a significantly higher increase in population size than control plots that were further away; the 2 detached control plots were unaffected (Figures 3b and 4). We observed an immediate and long-term use of the artificial pools by *A. femoralis* males for tadpole deposition. The gradual effect corresponds to the pattern expected from a new population source at the pools. Assuming a uniform dispersal of postmetamorphic juvenile frogs from the pool array, this is a first indicator that the population growth mainly reflected an increase in autochthonous recruitment.

### Sex differences and survivors

Both sexes responded with a similar increase to the added tadpole-rearing sites (Figure 3c). The results changed when we examined patterns for individuals that survived for more than a year. Although pool installation increased relative female survival, relative male survival decreased posttreatment (Figure 3e), although the interaction effect in the GLM was only marginally significant. There was a significant differential effect of the pools on survival across distance from the treatment plot. Although overall population size increased in the treatment plot, relative survival almost halved. At the same time, relative number of survivors in all control plots increased slightly (Figure 3b,d).

The effect of the resource supplementation on relative survival can be explained by a synopsis of findings from previous studies, and the 2 interaction effects of “treatment × distance” and “treatment × sex.” First, females are not aggressive and do not compete with one another for space or other resources (Ringler et al. 2009). For males, territory possession is a prerequisite for reproductive success (Ursprung et al. 2011a), and males vigorously defend territories against calling intruders (Narins et al. 2003). In a previous study, we identified a pronounced male year-to-year displacement away from tadpole pools (Ringler et al. 2009). This movement resulted from the space competition between male recruits and previous territory owners. The emergence of juveniles from the artificial pools likely caused a similar, radial expulsion of surviving males. The overall decrease in male survival after pool installation then likely reflects the higher stress from competition after increased recruitment, whereas the less aggressive females were unaffected. The differential survival of males was also evident in the operational sex ratio: it changed significantly toward a less male-biased ratio after pool installation.

### Pedigree analysis

Our pedigree estimation corroborated the findings of the demographic analysis. All parents, sampled and simulated, of every offspring generation, respectively, formed a contiguous cluster of males and females that were connected via their offspring. There was not a single mother–father dyad with both partners simulated, where neither of them had offspring with at least one further, sampled partner as well. Such otherwise unconnected, simulated parent pairs would have been an indicator for immigrating offspring whose parents had not been part of the reproductive community of the study area. Accordingly, our population was a source population before and even more so after the resource supplementation. The lack of a significant correlation between reproductive output and pool distance indicates that all individuals benefited equally, and that no artificial bottlenecks were created by distance effects on reproductive output.

The changes in reproductive success differed in the sexes. Males increased their output by having significantly more offspring per male after the resource supplementation. Females, in turn, even had a slight (but not significant) decrease in their number of offspring; this was counteracted by a change in the sex ratio of actual reproducers, probably reflecting the similar significant shift in the operational sex ratio. Thus, a reduced male survival, relative to females, accompanied the increased per-capita reproduction of males. In turn, the higher survival rate of females increased their relative number, but females had no increased per-capita reproduction. The observed patterns relate directly to *A. femoralis* reproductive behavior. Males did benefit individually from the artificial pools because they are responsible for parental care in the form of tadpole transport. They can, therefore, directly influence their reproductive success by choosing favorable water bodies (i.e., no desiccation, few predators, and sufficient food) for their offspring. Males probably repeatedly use suitable pools, once they have found them. Females exhibit high levels of polyandry (Ursprung et al. 2011a) and apparently mate with all males in their vicinity (Ringler et al. 2012). They leave their partners immediately after clutch deposition (Montanarin et al. 2011) and thus cannot assess how well males transport the offspring. This prevents females from increasing their individual reproductive success by choosing those males that used artificial pools and thus boosted their reproductive success. Nonetheless, the higher number of reproductively successful females, resulting from the change in the operational sex ratio, still caused an overall increase in female reproductive output (and thus the overall increase in population size).

### Conservation aspects

Our results have direct implications on conservation and management actions. They corroborate the notion of Zimmerman and Bierregaard (1986) and Laurance et al. (2002) that effective conservation of Neotropical rainforest anurans has to incorporate the effective protection and management of other species that shape anuran habitats, such as peccaries. This calls for targeted action because populations of *T. pecari* are declining dramatically across the entire range (Altrichter et al. 2012). Neglecting such ecosystem engineers clearly jeopardizes any narrower attempt to protect dependent species.

More amphibian-focused, technical conservation measures, such as suggested by Shoo et al. (2011), might help endangered amphibian populations by supporting their reproduction. Supplementing artificial aquatic sites might be especially helpful for populations affected by climate change and drying natural water bodies. Our findings—that higher rainfall in the preceding hydrological year significantly boosted the population size in the following year as a result of increased recruitment—support this notion.

Another threat which is facing dendrobatids, namely illegal and uncontrolled poaching for the pet trade, might be alleviated by artificial resource supplementation. Trade restrictions or bans would concurrently ameliorate the poaching problem and disease transmission (Kriger and Hero 2009). But this may not be realistic (Garner et al. 2009) and would also deprive indigenous people of one of their natural resources. A sustainable in situ management of dendrobatids desired by the pet trade might benefit forest owners, frogs, and the forests both inhabit. In situ breeding could be facilitated by supplementing reproductive resources to increase the yield of managed populations.

## FUNDING

This work was funded by the Nouragues Grant from the French Centre National de la Recherché Scientifique (CNRS) to M.R. and supported through the Austrian Science Fund (FWF, project: 18811, PI: W.H.). M.R. and E.R. were supported by a Forschungsstipendium of the University of Vienna and by the Austrian Science Fund (FWF, project: 24788-B22, PI: E.R.) and E.R. was funded by a DOC-fFORTE scholarship and a L’Oréal Austria fellowship “For Women in Science” from the Austrian Academy of Sciences.
